# Intention to Purchase Milk Packaged in Biodegradable Packaging: Evidence from Italian Consumers

**DOI:** 10.3390/foods10092068

**Published:** 2021-09-02

**Authors:** Antonella Cammarelle, Rosaria Viscecchia, Francesco Bimbo

**Affiliations:** Department of Agriculture, Food, Natural Resource and Engineering (DAFNE), University of Foggia, 71122 Foggia, Italy; rosaria.viscecchia@unifg.it (R.V.); francesco.bimbo@unifg.it (F.B.)

**Keywords:** sustainable packaging, biodegradable, milk, whey, consumer’s intention to purchase, consumer’s willingness to pay

## Abstract

The dairy industry generates large volumes of liquid waste that can be used to produce biopolymers, potentially employable for the creation of milk biodegradable bottles. In that regard, this paper aims to explore the consumers’ intention to purchase sustainable packages, as well as to assess the willingness to pay for it considering renewable packages made using organic waste feedstocks from the dairy industry (e.g., whey) and plant-based material (e.g., corn, sugarcane, etc.). To reach the stated objectives, we collected individual-level information (e.g., age, gender, education, income) from a convenient sample of 260 Italian consumers and a modified version of the Theory of Planned Behavior estimated using a structural equation model. Findings show that attitudes and perceived behavioral control are the most important drivers of the consumers’ intention to purchase sustainable packages. Finally, statistics show that respondents slightly prefer to purchase products packaged using plant-based biodegradable material, as well as most of the respondents show a low willingness to pay for milk offered in biodegradable packaging, regardless of the raw material used. Then, policymakers and companies should invest in educational/informational campaigns pointing out the beneficial effects on the environment from the purchase of foods in sustainable packaging. This may potentially increase the consumers’ intention to purchase, as well as their willingness to pay for plant-based and dairy whey-based packages by increasing the sustainability of the dairy supply chain.

## 1. Introduction

The dairy supply chain annually produces milk products for approximately six billion people worldwide, resulting in one of the most important sectors in the food industry [1]. Nowadays, Europeans have one of the highest per capita consumptions of dairy products worldwide, which is approximately 150 kg per year per capita [1,2]. Moreover, milk production has increased by 59% reaching 852 Mt over the last three decades [1,3], and it is forecasted to rise by nearly 15 Mt per year by 2030 [4]. The rise in milk production is currently spurred by the population growth and milk consumption in developing countries [4,5,6]. According to the International Farm Comparison Network (IFCN) (2018), 1.2 billion more consumers will demand milk products by 2030 [6].

Despite widespread dairy products consumption, 20% of it is annually lost or wasted along the whole food supply chain (FSC) worldwide [7]. Dairy products loss and waste mostly occur at the consumption [7,8] and manufacturing level in industrial countries [9,10] Dairy whey represents the main by-product that is lost or wasted [8,11,12]

The European annual production of dairy whey is estimated at 50 Mt and 40% is discarded instead of being recycled or reused, contributing to make the dairy supply chain one of the most unsustainable of the food sector [9,12]. Proper whey disposal is the most important environmental problem for the dairy industry [13,14], due to its both large volume and high organic content [10,15]. However, dairy whey contains nearly 55% of the milk nutrients [16], representing a potential resource to produce added-value products from its reuse (e.g., food supplements) [9,10]. In line with the “waste management hierarchy”, dairy whey can be potentially reused as an input for other production systems [17,18].

From a circular economy prospective, a promising feasible solution to increase the sustainability of the dairy supply chain could be the use of the whey, an organic waste, for the production of innovative biopolymers such as poly(butylene-co-adipate terephthalate) (PBAT), polyhydroxyalkanoates (PHA), polylactic acid (PLA), and polyvinyl acetate (PVA) for food packaging applications [14,19,20,21]. These polymers are completely bio-based, biodegradable and their barrier properties are comparable to the conventional petroleum-derived alternatives [22]. Moreover, PHA is UV-resistant and oxygen-impermeable (fundamental properties for food packaging) and it is employable for the production of bottles and water-resistant film [13,14,19]. 

The above-mentioned packaging materials could be suitable solutions to mitigate the removal and disposal problems of the common packages for liquid dairy products, such as HDPE (high-density polyethylene) bottles and Tetra Pak systems [23]. Indeed, in Europe only 10–15% of the 2 million tons of HDPE bottles, commonly used for UHT (ultra-high temperature) milk, are currently recycled [23]. Further, the adoption of dairy whey-based packages will help to reduce the dependence on fossil resources, their price increase, and to further improve the sustainability of the dairy supply chain [22,24]. 

Alternative sustainable packages than whey-based ones made by organic waste feedstocks are plant-based ones (e.g., corn, sugar cane etc.), which currently represents the most efficient option for the production of bioplastics [22]. Besides that, their production costs are still high, representing the main barrier for companies to adopt these [14]. For instance, bioplastics are generally more expensive than conventional ones [24], and according to the BIOBOTTLE project report, the cost of fresh milk in large plant-based biodegradable bottles increases less than 10% in comparison with the current packages [23]. Instead, the use of the whey could help to reduce the unit price for the production of the biopolymer by almost 23% but its development is still in an early stage [25]. 

Nowadays, food and packaging industries are joining efforts to use biodegradable materials, in order to reduce the amount of plastic waste sent to landfills [10,26]. However, innovations in the food sector, including packaging, are successful only if accepted by consumers [27,28]. Therefore, the introduction of new packages into the market may result in profit for food companies as long as consumers accept them and are willing to pay for such innovative solutions. 

In this study, we explore consumers’ intention to purchase and willingness to pay for milk packaged in dairy whey-based polymer, or organic waste feedstocks, and plant-based ones (e.g., corn, sugarcane, etc.). The consumer adoption of milk packaged using such polymers would increase the sustainability of the dairy supply chain, especially whether the dairy whey-based polymer is preferred over plant-based one. In our study, we employ a sample of Italian consumers, as well as a modified version of the theory of planned behaviour. To the best of our knowledge, there is no study available investigating Italians’ intention to purchase, and preferences for, well-defined sustainable packaging (dairy whey and plant-based packages). Existing studies, indeed, have focused on consumers sampled outside Italy, for which test their preferences towards undefined sustainable packages.

### Literature Review on Individual Driver of Sustainable Consumption and Theoretical Framework

Studies on consumers’ sustainable choices are mostly focused on the Theory of Planned Behavior (TPB) [29]. This theory assumes that the intention to perform a behavior is influenced by attitudes, subjective norms, and perceived behavioral control [29]. 

Attitudes towards a specific behavior represent the personal favorable or unfavorable evaluation of performing that behavior [30]. The reviewed studies pointed out as consumers with positive attitudes toward preserving the environment were more willing to consider undefined sustainable packaging in their purchase decisions [31,32]. Moreover, studies showed that consumers with positive attitudes toward undefined sustainable packaging also reported strong positive attitudes in favor of recycling [31,33,34,35]. Indeed, consumers with pro-environmental attitudes were more likely to adopt multiple sustainable behaviors, with respect to different topics, such as recycling [36,37,38,39], waste management [40,41,42,43,44], energy consumption [45], transport use [46], the purchase of green products [47]. Therefore, consumers that consider the importance of the correct packaging disposal at the end of its useful life will also be the ones willing to purchase sustainable products [31,32]. 

However, positive environmental attitudes are not able to predict the behavior if social norms are not considered [48]. The importance that society places on environmental issues plays an important role in explaining sustainable consumption behavior as well [31]. Specifically, the subjective norm is defined as the personal perception of the social pressure to behave in a certain way or not [29]. Then, consumers who perceive high social pressure to preserve the environment, by the use of sustainable packaging with undefined material or disposing packages in a correct way, could be also more willing to purchase foods packaged in sustainable solutions [31,36,48,49,50]. 

Reviewed studies showed that sustainable consumption is also influenced by the perceived behavioral control [31,32,51,52]. It represents the individual perception of difficulty or simplicity to perform a specific behavior [29]. In this context, it is defined as the personal view of the capacity for contributing to solving environmental issues [31,51,52]. Then, a consumer’s purchase decision can be affected by their belief that his or her actions or environmental practices (e.g., recycling) could help to protect the environment. Indeed, the stronger the individual’s perceived behavioral control, the greater the consumer’s intention to purchase food packaged in sustainable packages. 

Furthermore, studies in the literature confirmed the relationship on sustainable consumption is mediated by the consumer’s awareness of environmental issues [31,53]. Specifically, the awareness of the risks for human health, due to environmental pollution, is considered one of the most important drivers of the consumer’s intention to purchase sustainable products [54]. Furthermore, the consumer’s awareness about the causes affecting environmental problems (e.g., wrong packaging disposal) is also considered significant in explaining a consumer’s sustainable purchase decisions [54]. 

**Figure 1:**
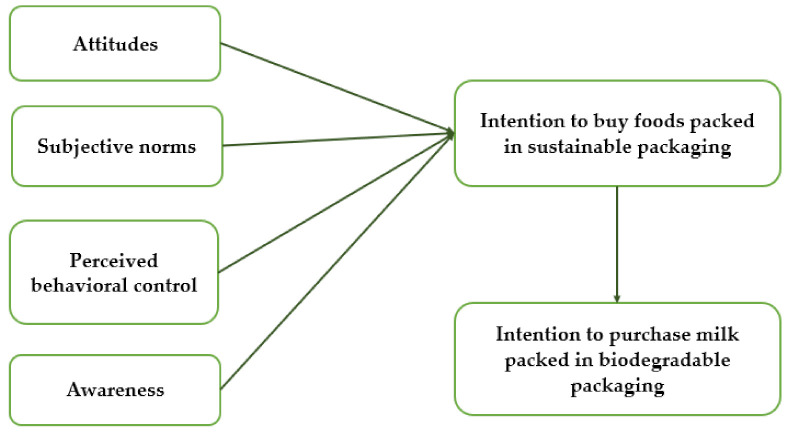
Determinants of the consumer’s intention to buy foods packed in sustainable packaging and intention to purchase milk packed in biodegradable packaging.

Finally, the intention to buy foods packed in sustainable packaging will traduce in reality only if the abstract intention is linked to a more concrete goal to perform a specific behavior, such as purchasing milk packed in biodegradable packaging, as also supported by the Goal Implementation Theory [55,56,57,58]. The most popular Geographic areas, of the studies briefly described above, were Northern Europe, the U.S., China and other developing countries, such as India. In Southern Europe, there were two; one in Portugal and the other in Spain. No evidence was found for Italian consumers. Then, Figure 1 shows the proposed empirical framework and the link between all the factors described above.

## 2. Materials and Methods

### 2.1. Participants and Design

Data were collected by means of a web-based survey conducted in April 2020 in Italy. The survey was targeted to Italians over 18 years old, who are responsible for the food shopping in their household and who purchase milk at least once in a month. Before starting the survey, a brief explanation of biodegradable packaging was provided to respondents, as reported in Appendix A—Table A1. In this study we used a convenient sample composed by 260 respondents recruited through the main social networks (e.g., Facebook, LinkedIn, WhatsApp). The sample is made up of Italian consumers equally distributed between North, Center, South and Islands recruited via online web survey and who did not receive any compensation for participating in the study. Most of the respondents were female (69.6%) with an average age of 35.8 (SD = 11.7). The sample was highly educated, since 32.3% of consumers had completed high school and 66.6% had completed higher education. Most of the participants were employed (53.1%) with a family monthly income of between EUR 1001–3000 (46.5%). Households were composed of three members (M = 3.4; SD = 1.2) with an inconsistent number of children under 14 years old (M = 0.4; SD = 0.7). Finally, the analysis of the milk shopping habits is reported in Table 1 showing that most of the respondents usually buy Ultra High Temperature (UHT) milk (51.5%), two or more times in a week (32.2%). Most of them usually buy low-fat milk (86.9%) packaged in Tetra Pak^®^ (55%), even if the plastic option is also very common by respondents (43.8%). Finally, most of them usually buy 1 L packs of milk (81.2%), at the unit price between €1.01 and €1.50 (37.7%), and usually buy up to ten packs in a month (67%).

### 2.2. Measures

The questionnaire contained measures of attitudes, subjective norms and perceived behavioral control toward sustainable food packaging, awareness of environmental issues, and its link with human health, intention to buy foods packed in sustainable packaging, intention to purchase and to pay for milk packed in biodegradable packaging, and socio-demographics. Moreover, the survey also contains questions about the milk shopping habits, as shown in the Appendix A—Table A2.

In relation to the TPB constructs, plus awareness, respondents were asked to indicate their agreement or disagreement to some statements scored on a seven-point Likert item scale ranging from “totally disagree” (1) to “totally agree” (7).

Following the TPB [29], a measure of general attitudes toward sustainable food packaging was used, assessed with 3-items scale: “Food packaging waste has negative consequences for the environment”, “All food packaging should be environmentally friendly (e.g., biodegradable) to reduce their environmental impact” and “All food packaging should be environmentally friendly, even if that requires a small charge in its price”. These statements were developed in accordance with the TPB and with the prior literature on sustainable consumption [31,54].

Subjective norms were composed by 2-items scale: “People who are important to me (e.g., family, friends) believe that it is very important to properly dispose of food packaging” and “The most important persons to me (relatives and friends) believe that buying food products packaged in sustainable packaging (e.g., biodegradable) is a behavior that helps to preserve the environment” [31,54]. 

Individual perceived behavioral control was assessed with 2-items scale: “My food packaging disposal choices have a direct impact on the environment” and “Choosing to buy food products packaged in sustainable packaging (e.g., biodegradable) contributes to solving environmental problems” [31,54].

Moreover, consumers’ awareness of environmental issues was measured with a 2-items scale: “My health and well-being are strongly related to environmental quality” and “Food packaging waste is one of the most important environmental issues” [54]. 

The intention to buy foods packaged in sustainable packaging was measured using 3-items scale: “I intend to purchase food packaged in sustainable packaging in the next months”, “I plan to purchase food packaged in sustainable packaging in the next months” and “I want to purchase food packaged in sustainable packaging in the next months” [59]. 

Finally, to measure the intention to purchase milk packaged in biodegradable packaging, respondents were asked to indicate their intentions, with a 7-point Likert item scale ranging from “totally not willing” (1) to “totally willing” (7), related to this statement: “Are you willing to purchase milk packaged in biodegradable packaging?”. 

Last, the mean value was calculated for all the constructs measured by using multiple items scale, as shown in Appendix A—Table A3. The latter also shows the correlations between all the variables considered in the proposed empirical framework. 

### 2.3. Estimation Method

The conceptual model proposed by the authors was tested performing the Structural Equation Modeling (SEM), through the use of STATA 16.0 software (StataCorp LLC, College Station, TX, USA). This analysis helps to identify the magnitude and direction of the relationships between the variables. To verify the goodness-of-fit of the SEM model, the chi-square test and the incremental goodness-of-fit indices were estimated. According to Iacobucci [60], the model works well when the Chi-Square is not significant [60]. Moreover, “the Root Mean Square Error of Approximation (RMSEA) values < 0.05 constitute good fit, values in the 0.05 to 0.08 range acceptable fit, values in the 0.08 to 0.10 range marginal fit, and values > 0.10 poor fit [61], for both the Comparative Fit Index (CFI) and Tucker-Lewis Index (TLI) values > 0.95 constitute good fit and values > 0.90 acceptable fit [62,63] and Standardized Root Mean Square Residual (SRMR) should be lower than 0.08 [59,64]. 

## 3. Results

The results obtained by testing the empirical framework are shown in Table 2. The model showed an acceptable goodness of fit considering that the RMSEA was between 0.05 and 0.08 range; both the CFI and TLI values were higher than 0.95 and the SRMR value was extremely lower than 0.08. Overall, explained variance was equal to 46.07%.

Results from the model showed that the individual intention to buy foods packaged in sustainable packaging was a good predictor of a consumer’s intention to purchase milk packed in biodegradable packaging (0.555 *p* < 0.001). With respect to the determinants of the intention to assume a more ecological purchase behavior, all the variables concerning the TPB were significantly and positively related to the individual’s intention to buy foods packaged in sustainable packaging. In detail, attitude towards sustainable packaging was the most important driver of the personal intention to perform the behavior (0.468 *p* < 0.001), followed by perceived behavioral control (0.287 *p* < 0.001) and subjective norms (0.100 *p* < 0.05). This finding was also consistent with the correlation matrix shown in Appendix A—Table A3, third column, reporting the correlation index between the attitudes and the intention to buy foods packaged in sustainable packaging as the highest. A consumer’s awareness of environmental issues was also an important predictor of the individual intention, with magnitude of the coefficients equal to 0.138 (*p* < 0.01). However, the socio-demographics characteristics, such as, age, gender, and education level, inserted as control variables, did not affect the consumer’s intention to buy foods packaged in sustainable packaging.

### Willingness to Purchase and to Pay for Milk Packed in Biodegradable Packaging

The results showed that almost the totality of the respondents (92%) who intended to buy foods packaged in sustainable packaging were also willing to purchase milk packed in biodegradable packaging, in order to improve the environmental wellbeing (58.6%), as shown in Appendix A—Table A4. However, consumers mostly preferred the use of plant-based raw materials (e.g., corn, sugarcane etc.) (55.65%) rather than the use of organic waste feedstocks (e.g., whey) (44.35%). Indeed, most of the respondents disliked the idea to use wastes to create food packaging (*n* = 47), as well as using organic waste feedstocks (e.g., whey) was perceived potentially risky for human health (*n* = 41). Finally, most consumers were also willing to pay 1–5% more for milk packed in biodegradable packaging made from organic waste feedstocks (43.40%), as well as from plants (51.88%), as shown in Table 3. A large portion of respondents, equal to 28.87% and 30.83%, would also be willing to pay 6–10% more for organic waste and plant-based packaging for milk, respectively. Only 7.95% of consumers were not willing to pay a premium price for milk packaged in biodegradable packaging.

## 4. Discussion

The present study investigated they type of factors that can drive consumers toward more ecological purchase decisions through an extended TPB model. This appears to be be relevant in explaining the consumer’s intention to buy foods packaged in sustainable packaging. 

The results highlighted that attitude was the most important predictor of the personal intention to behave in a pro-environmental way. This finding was supported by Van Birgelen et al. [31] who, in their study on German consumers (*n* = 176), pointed out that respondents who showed positive attitudes toward preserving the environment were more willing to consider sustainable packaging in their beverage purchase decisions [31]. This result was consistent with the study of Mobrezi and Khoshtinat [65], on Iranian consumers (*n* = 279), showing that the intention to buy undefined sustainable products increased by the rising of positive attitudes toward the environment [65]. Attitude about using sustainable products had a positive and a significant association with the behavioral intention for other studies present in literature [66,67,68]. 

In our research, perceived behavioral control was the second most important driver of the consumers’ intention to purchase foods packaged in sustainable packaging. Then, Italian respondents who recognized the importance of assuming more ecological purchasing behaviors were also more likely to buy sustainable food packaging, and thus, for milk. Therefore, in our study, consumers who believed that their actions or environmental practices, such as purchasing sustainable packaging and disposing used packaging in a correct way, had positive impacts on the environment were also willing to consider sustainable packaging for foods in their purchasing decisions, as reported in Van Birgelen et al. [31]. The perceived behavioral control was also found to be positively and statistically related to the consumer’s intention to purchase undefined sustainable packaging by Auliandri et al. [69], which investigated young Indonesian consumers (*n* = 276) [69]. However, perceived behavioral control was found, in Auliandri et al. [69] study, to be the fourth driver of consumer’s intention to purchase sustainable packaged goods, after environmental concerns, willingness to pay, and subjective norms, that scored higher magnitude [69]. Consumers’ cross-cultural differences, as well as differences in research design can explain such contrasting findings. Similar results were also found by many other studies present in literature [31,54,66]. 

Additionally, subjective norms emerged to be positively and significantly related to the intention to assume sustainable purchase decisions. This could mean that what others believe is important is able to influence the individual behavior. This result was supported by Van Birgelen et al. [31] and Auliandri et al. [69] highlighting how the social perception about sustainable products and their importance for improving environmental wellbeing encourage consumers to buy foods packaged in sustainable packaging [31,69]. Contrasting findings were found by Chen and Hung (2016), in their study on Chinese consumers (*n* = 406), and Mobrezi and Khoshtinat [65] showing as the role of social pressure, exercised by relatives and close friends, is not significantly related to the intention in purchasing undefined sustainable products [65,66].

Furthermore, the results from our research showed that Italian consumers with high environmental consciousness, as well as being aware about the risks for human health, due to the environmental pollution, were also more likely to consider sustainable packaging in their purchase decisions. This finding is consistent with many studies present in the literature, which suggest that the consumers’ intention to buy sustainable products usually increases by the rising of environmental concerns [31,54,65]. 

Finally, socio-demographics characteristics such as, age, gender and the education’s level, inserted as control variables, were found to not be significant in explaining the Italian consumer’s intention to buy foods packaged in sustainable packaging. This result was supported by Suki [70], in a study on Malaysian consumers (*n* = 200), who confirmed that respondents’ demographics (e.g., gender, age) did not affect the consumer’s pro-environmental behavior [70]. Contrasting findings were found by Rokka and Uusitalo [48], who in their study on Finland respondents (*n* = 330), showed that sustainable packaging buyers are usually more likely to be female and older consumers. The level of education was not found to be significant in affecting consumers’ intentions to buy sustainable packaging [48]. This could be due to the greater attention that the media has given on environmental issues thus managing to involve consumers with lower levels of education. 

Once the drivers of the personal intention to assume more ecological purchase decisions are identified, this research aimed to analyze the Italian consumer’s intention to buy milk packaged in biodegradable packaging, as well as to investigate how the respondent’s willingness to pay varies from different raw materials, such as, organic waste feedstocks (e.g., whey), as well as plant-based (e.g., corn, sugarcane etc.).

The results showed that almost the totality of the interviewed were willing to purchase milk in biodegradable packaging to improve the environmental wellbeing. This finding was supported by Koutsimanis et al. [71] who, in their study on North Americans (*n* = 292), showed that bio-based packaging for fresh foods was the most preferred option by consumers [71], rather than the conventional ones. Arboretti and Bordignon [72], in their study on Italian and Austrian respondents (*n* = 205), found that the biodegradability was the favored food packaging attribute for the consumer final choice [72]. Moreover, many studies in the literature suggested that perceived benefits were the significant predictors of the consumer’s intention to purchase sustainable packaging. Then, the protection of the environment, as well as the reduction of the risks for human health were the main reasons for individual pro-environmental behavior [73,74,75]. 

Further results of our study highlighted that the plant-based feedstock (e.g., corn, sugarcane etc.) was the favored raw material for milk biodegradable packaging, although a great share of respondents chose the organic waste option (e.g., whey). In this regard, perceived risk for human health was one of the principal reasons for rejection of biodegradable packaging made from organic waste feedstock. Similar results were found by Magnier et al. [76] who, in their study on Dutch consumers (*n* = 258), found that the risks of contamination negatively influenced the consumer’s purchase intention of products made from recycled ocean plastics [76]

Finally, most of the respondents in our research were also willing to pay a premium price for milk packaged in biodegradable packaging regardless of the origin of the raw material used. This finding was consistent with Grebitus et al. [77] who, in their study on North Americans (*n* = 109), found that consumers who received pro-environmental guidance appeared to be willing to pay a higher price for both plant-based and recycled plastics [77]. The majority of the participants were college students (70.6%) with 37.6% self-identifying as female [77]. Similar results were observed by Neil and Williams [78], in a study on USA consumers (*n* = 229), showing that most of the respondents (81%) were willing to pay a premium price for sustainable packaging [78]. Specifically, if consumers perceived the returnable glass bottle for milk to be more environmentally friendly than plastic, they were willing to pay 26.78 cents more [78]. The average responding consumer was between 30 and 45 years of age, with two or three people living in the household [78]. This finding was also confirmed by 67% and 86% of the respondents of surveys conducted in Germany (*n* = 176) and Sweden (*n* = 712) in which consumers were found to be willing to pay at least $0.13 more for environmentally packaged beverage and 6% more for undefined sustainable packaging, respectively [31,79]. In these two studies most of the participants were female with a high education’s level [79]. Therefore, being female, young, and highly educated was associated with a positive, albeit marginal, willingness to pay for sustainable packages.

## 5. Conclusions

The present work provides relevant information about the factors able to drive consumers toward more sustainable purchase decisions. The results show that pro-environmental attitudes, perceived control over the individual actions (e.g., recycling), the social pressure to preserve the environment, as well as a consumer’s awareness for the environmental issues are able to explain the personal intention to purchase sustainable packaging for foods, and thus, for milk. Furthermore, the findings highlight that consumers mostly prefer plant-based (e.g., corn, sugarcane etc.) biodegradable packaging for milk. Indeed, 55.65% of respondents prefer plant-based biodegradable packaging for milk, while the remaining 44.35% preferred dairy whey-based packaging. This is because the use of organic waste feedstocks (e.g., whey) for food packaging applications is perceived as potentially risky for human health by some respondents. However, regardless of the renewable origin of the raw material, consumers are willing to pay 1–5% more for milk within sustainable packaging. 

Given the absence of studies on this topic and specifically on the consumer’s intention to purchase a defined food product (e.g., milk) packaged in sustainable alternatives, these results may fill the gap in literature for the Italian market contributing to improve the knowledge in this field. Then, these findings come with important policy and marketing implications. Policymakers and companies may develop informational and educational campaigns to raise the level of awareness about the negative impact of packaging waste on the environment, as well as on human health, which may have an important role in supporting behavioral changes toward more sustainable purchasing options. Additionally, companies may also promote with marketing campaigns the use of organic waste feedstocks to create biodegradable packaging. Such a message should focus on increasing the consumer’s knowledge about the use of whey as totally safe food contact material, considering also that this by-product of the dairy industry is commonly used to produce food supplements (e.g., whey proteins). In this regard, policymakers should encourage, with incentive based-policy (e.g., tax relief), companies to reuse the whey for the production of value-added products to increase the efficiency of the dairy industry and adopt closed-loop recycled systems. 

Finally, some limitations should be considered to evaluate our results. First, given the sample size, these findings cannot be generalized to the Italian population, as well as to other geographical contexts. Moreover, the sample is mostly composed of respondents with a high education’s level which could significantly affect our results, specifically with reference to consumers’ attitudes, their perceived behavioral control, subjective norms, as well as their awareness. Second, the total variance explained by our model, equal to around 46%, could mean that factors included in our version of the TPB are not able to explain all the potential drivers able to guide consumers toward more sustainable purchasing behaviors. Furthermore, the factors used in the model showed a positive and a significant effect on the consumer’s intention to purchase sustainable packaging. However, the wording and number of the items proposed by the authors may affect the importance rank of individual factors to adopt pro-environmental behaviors such as purchase milk in dairy whey and plant-based packaged milk. Moreover, results show that consumers willing to purchase foods packaged in sustainable packaging will have a 50% chance to also choose milk in biodegradable containers. This could highlight a difficulty for consumers to change their purchasing habits also in relationship with the packaging. Indeed, most respondents usually buy milk in Tetra Pak) that could be considered by consumers as an existing sustainable option over plastic that can also ensure food safety and shelf-life. 

Therefore, future research should be focused on mitigating the limitations listed above using a larger and more representative sample of the Italian population. Further, the selection of different or a larger number of items to capture factors included in our TPB, as well as accounting for environmental situation (e.g., supermarket) or emotional and unconscious stimuli, could offer more granular and robust evidence on the drivers of consumers’ intentions in purchasing milk in biodegradable packaging and paying a premium price for that milk. 

## Figures and Tables

**Table 1:** Milk shopping habits (*n* = 260).

Categorical Variables	Sample%
**Milk shopping frequency**	
Once in a day	5.8
Two or more times in a week	32.3
Once in a week	29.6
Two or more times in a month	19.2
Once in a month	13.1
**Milk type**	
Fresh pasteurized milk	32.7
High temperature pasteurized milk	2.3
Microfiltered milk	6.9
UHT milk	51.5
I don’t know	6.5
**Fat content**	
Whole milk	19.6
Low-fat milk	86.9
Skim milk	13.1
**Type of packaging**	
Plastic	43.8
Glass	1.2
Tetra Pak	55.0
**Package’s size**	
0.5 lt	13.1
1 lt	81.2
1.5 lt	5.8
**Number of packages in a month**	
0–5	33.5
6–10	33.5
11–15	18.1
16–20	7.3
>20	7.6
**Price of a package**	
€0–€0.5	23.1
€0.51–€1.00	22.3
€1.01–€1.50	37.7
€1.51–€2.00	13.8
>€2.00	3.1

**Table 2:** The structural model of the consumer’s intention to buy foods packed in sustainable packaging and then to purchase milk packed in biodegradable packaging.

Parameters	Intention to Purchase Milk Packed in Biodegradable Packaging
	Coefficient
Intention to buy foods packed in sustainable packaging	0.555 ***
	Intention to buy foods packed in sustainable packaging
Attitudes	0.468 ***
Subjective norms	0.100 **
Perceived Behavioral Control	0.287 ***
Awareness	0.138 *
Age	−0.002
Gender	−0.100
Education’s level	0.167
Indexes of goodness-of-fit	
R^2^	46.07%
Likelihood Ratio 𝜒 2 (6)	14.01 *p*-value < 0.05
RMSEA	0.072
CFI	0.969
TLI	0.922
SRMR	0.020

Note: *, ** and *** indicate 10, 5, and 1 percent significance levels, respectively.

**Table 3:** Consumers’ willingness to pay a premium price for milk packaged in biodegradable packaging.

Willingness to Pay a Premium Price	Plant-Based Feedstocks		Organic Waste Feedstocks		TOTAL	
	*n*	%	*n*	%	*n*	%
0% more	11	8.27	8	7.55	19	7.95
1–5% more	69	51.88	46	43.40	115	48.12
6–10% more	41	30.83	28	26.42	69	28.87
11–15% more	8	6.02	15	14.15	23	9.62
16–20% more	4	3.01	9	8.49	13	5.44
TOTAL	*133*	*100*	*106*	*100*	*239*	*100*

## Data Availability

Data sharing not applicable.

## Appendix A

**Table A1:** General information about biodegradable packaging.

Technology	Description
Biodegradable packaging	“Biodegradable materials are materials that can be broken down by microorganisms (bacteria or fungi) into water, naturally occurring gases like carbon dioxide (CO_2_) and methane (CH4) and biomass (e.g., growth of the microorganism population). Biodegradability depends strongly on the environmental conditions: temperature, presence of microorganisms, presence of oxygen and water. So both the biodegradability and the degradation rate of a biodegradable packaging may be different in the soil, on the soil, in humid or dry climate, in surface water, in marine water, or in human made systems like home composting, industrial composting or anaerobic digestion [Van den Oever et al., 2017]”. Finally, biodegradable packaging can be bio-based which means that the material or product is totally or partly derived from biomass. Today, bio-based and biodegradable packaging are mostly made of carbohydrate-rich plants such as corn or sugarcane, so called food crops or first generation feedstock. However, this kind of packaging can also be made from ligno-cellulosic feedstock such as plants that are not eligible for food and feed production or from organic waste feedstocks (e.g., whey) [European Bioplastics, 2018; ENEA, 2018].

**Table A2:** The questionnaire’s structure.

Section	Questions	Response Variable	Response Option
Milk Shopping Habits	Milk shopping frequency	Multiple Choice	Once in a day; two or more times in a week; once in a week; two or more times in a month; once in a month.
	Type of milk	Multiple Choice	Fresh pasteurized milk; high temperature pasteurized milk; microfiltered milk; UHT (Ultra High Temperature) milk; I don’t know.
	Fat content	Multiple Choice	Whole milk; low-fat milk; skim milk.
	Type of packaging	Multiple Choice	Plastic; Glass; Tetra Pak.
	Package’s size	Multiple Choice	0,5 lt; 1 lt;1,5 lt; other.
	Number of packages in a month	Open-ended	numeric
	Price of a package	Open-ended	numeric
Theory of Planned Behavior	Awareness, Attitudes, Subjective norms, Perceived Behavioral control, Intention to buy foods packed by sustainable packaging	Likert scale	7-point Likert scale ranging from 1 (totally disagree) to 7 (totally agree)
Intention to purchase and to pay for milk packed in biodegradable packaging	Intention to purchase	Likert scale	7-point Likert scale ranging from 1 (totally not willing) to 7 (totally willing)
	Renewable origin of milk packaging	Dichotomous	plant-based feedstocks (e.g., corn, sugarcane etc.); organic waste feedstocks (e.g., whey).
	Reason of the intention to purchase	Multiple Choice	Improvement of the environmental wellbeing; reduction of the dependence on fossil resources; disposing of the package with organic waste; creation of biogas and compost from the industrial composting; other.
	Reason of rejection	Multiple Choice	Price increasing; mechanical characteristics inferior to traditional packaging; risks for human health; otherS.
	Willingness to pay a premium price	Multiple Choice	0% more; 1–5% more; 6–10% more; 11–15% more; 16–20% more.
Socio-demographics	Age	Open-ended	numeric
	Gender	Multiple Choice	Male; female
	Education’s level	Multiple Choice	Primary School; Middle school; High School; Bachelor’s degree; Master’s degree; Postgraduate (e.g., PhD, master)
	Occupation	Multiple Choice	Not employed/student/housewife; Retired; Blue-collars; White-collars; Managers; Self-employed
	Family monthly income	Multiple Choice	Up to EUR 1000; EUR 1001–3000; EUR 3001–5000; EUR 5001–7000; EUR 7001 and over
	Household size, Number of children (under 14 years old), Number of employed in family (excluding interviewed)	Open-ended	numeric

**Table A3:** Descriptive statistics and correlations (*n* = 260).

Variables	Mean	SD	1.	2.	3.	4.	5.	6.
1. Intention to buy foods packed in sustainable packaging	6.32	0.89	1					
2. Attitudes	6.44	0.65	0.58 *	1				
3. Subjective norms	6.96	1.06	0.40 *	0.39 *	1			
4. Perceived behavioral control	6.34	0.82	0.57 *	0.54 *	0.43 *	1		
5. Awareness	6.56	0.62	0.43 *	0.52 *	0.28 *	0.55 *	1	
6. Intention to purchase milk packed in biodegradable packaging	5.63	0.67	0.56 *	0.45 *	0.24 *	0.37 *	0.41 *	1

Note: * indicate 1 per cent significant levels, respectively.

**Table A4:** Reasons for the intention to purchase biodegradable packaging for milk (*n* = 239).

Reasons	N	%
1. Improvement of the environmental wellbeing	140	58.6
2. Possibility to reduce the dependence on fossil resources	47	19.7
3. Possibility to create biogas and compost from the industrial composting process	34	14.2
4. Reduction of time to devote to separate collection (disposal with organic waste)	15	6.3
5. No one of these reasons	3	1.2
